# Plasma glutamine levels in patients after non-elective or elective ICU admission: an observational study

**DOI:** 10.1186/s12871-016-0180-7

**Published:** 2016-03-10

**Authors:** Hanneke Buter, Andries J. Bakker, W. Peter Kingma, Matty Koopmans, E. Christiaan Boerma

**Affiliations:** 1 Department of Intensive Care, Medical Centre Leeuwarden, Leeuwarden, The Netherlands; 2 Department of Clinical Chemistry, Medical Centre Leeuwarden, Leeuwarden, The Netherlands

**Keywords:** Plasma glutamine levels, Intensive care unit, Elective surgery, Non-elective admission

## Abstract

**Background:**

A low plasma glutamine level at the time of acute admission to the intensive care unit (ICU) is an independent predictor of an unfavourable outcome in critically ill patients. The primary objective of this study was to determine whether there are differences in plasma glutamine levels upon non-elective or elective ICU admission. The secondary objective was to compare glutamine levels over time, and to determine correlations between glutamine levels and the severity of illness and presence of infection in ICU patients.

**Methods:**

We performed a single-centre observational study in a closed-format, 22-bed, mixed ICU. Plasma glutamine levels were measured at admission and every morning at 6.00 a.m. during the ICU stay. We aimed to include at least 80 patients per group. The study was approved by the local Medical Ethics Committee.

**Results:**

In 88 patients after elective surgery, the median plasma glutamine level at admission was significantly higher compared with that in 90 non-elective patients (0.43 mmol/l [0.33–0.55 mmol/l] versus 0.25 mmol/l [0.09–0.37 mmol/l], *P* = 0.001). During the ICU stay, plasma glutamine levels remained significantly higher in elective patients than in non-elective patients. There was a significant correlation between the APACHE IV score and glutamine levels (*R* = 0.52, *P* < 0.001). Moreover, backward linear regression analysis showed that this correlation was independently associated with the APACHE IV score and the presence of infection, but not with the type of admission.

**Conclusions:**

Plasma glutamine levels are significantly lower after non-elective admission compared with elective admission to the ICU. A considerable amount of elective and non-elective patients have decreased plasma glutamine levels, but this is not independently associated with the type of admission. In contrast to previous studies, we found that plasma glutamine levels were determined by the severity of illness and the presence of an infection.

**Trial registration:**

ClinicalTrials.gov, number NCT02310035.

**Electronic supplementary material:**

The online version of this article (doi:10.1186/s12871-016-0180-7) contains supplementary material, which is available to authorized users.

## Background

Glutamine plays an important role in cell homeostasis and metabolism because of its role in gluconeogenesis, nitrogen transport, and synthesis of proteins and nucleic acids [1, 2]. During critical illness, the use of glutamine exceeds the supply, and plasma and skeletal muscle pools of free glutamine are reduced. Rapidly dividing cells, such as enterocytes and immune-competent cells, are especially sensitive to glutamine depletion [2].

A low plasma glutamine level at the time of acute admission to the intensive care unit (ICU) is an independent predictor of an unfavourable outcome in critically ill patients [3, 4]. Previous studies have shown that 30–67 % of patients who were admitted to the ICU had a glutamine level below 0.42 mmol/l [3–6]. Patients with lower glutamine levels are older compared with patients with normal glutamine levels and are more often diagnosed as having shock at admission [3].

Major surgery induces a stress response that is characterised by hormonal release and inflammatory processes [7, 8]. This stress response resembles catabolic stress, which is found in other forms of critical illness. Whether inflammation that is caused by major elective surgery also leads to a decrease in plasma glutamine levels, comparable to a decrease during sepsis and other forms of acute critical illness, is unclear.

The primary objective of the study was to determine whether there are differences in plasma glutamine levels upon non-elective or elective ICU admission. Our secondary objective was to compare the course of glutamine levels over time between the two groups during the ICU stay, and to determine the correlations between glutamine levels and the severity of illness and presence of infection in ICU patients.

## Methods

### Study design

We performed a single-centre observational study in a closed-format, 22-bed, mixed ICU in a tertiary teaching hospital. We included consecutive patients who were admitted to the ICU from 26 April until 4 July 2014. Exclusion criteria were age younger than 18 years and the need for total parenteral nutrition to avoid intravenous glutamine supplementation. Patients were divided into two groups: those admitted to the ICU after elective surgery and those with non-elective admission (medical and acute surgery).

The study was approved by the local Medical Ethics Committee (Regional Review Committee for Patient-related Research, Medical Centre Leeuwarden, nWMO 48, 24 March 2014). Informed consent was waived under the condition that the glutamine measurements were performed from residue blood samples, taken as part of standard care. The study was registered in a public register (ClinicalTrials.gov, number NCT02310035).

### Setting

Blood samples were collected at admission and every morning at 6.00 a.m. during the ICU stay. According to protocol, all of the patients were equipped with a nasogastric tube. After non-elective admission, enteral nutrition was started within 6 h according to our local feeding protocol. In patients after elective surgery, the feeding protocol was started as soon as it became clear that patients would not leave the ICU on day 1. Enteral nutrition was started at 20 ml/h (Nutrison Protein plus Multi Fiber®; Nutricia Advanced Medical Nutrition, Danone Baby and Medical Nutrition B.V.), and every 6 h, the amount of enteral nutrition was increased to 20 ml/h until the desired amount was reached. In patients with gastric residue of more than 250 ml/6 h, the amount of feeding was not increased. A duodenal feeding tube was placed in case a patient did not receive 40 ml/h nutrition within 48 h after admission. The desired amount of enteral feeding was 25 kcal/kg/h and 1.2 g protein/kg/h for patients with a body mass index (BMI) between 18 and 30. In case of a BMI lower than 18 or greater than 30, a correction towards ideal body weight was performed [9, 10]. For patients with a BMI greater than 30, we used the weight that patients would have had with a BMI of 30 [10]. Enteral glutamine (Glutaperos®; GLNP Life Sciences BV, 27 g daily) was started on day 3 in patients without renal replacement therapy or liver failure.

### Glutamine measurements

Measurement of glutamine was performed with the Bioprofile 100 plus analyser (Nova Biomedical U.K., Cheshire, UK). This analyser was originally intended for use for monitoring metabolites and nutrients in cell culture systems, and was used in this study for investigational purposes to monitor glutamine levels in plasma samples (see Additional file 1).

### Statistical analysis

There are no data available on plasma glutamine levels after elective surgery. Therefore, a true power calculation was not applicable. Consequently, we estimated a number of at least 80 patients per group to obtain a difference between the two groups. Descriptive statistics were used to summarise the study population. Data are shown as the mean with standard deviation or median with interquartile range, depending on the normality of the data. Differences between groups were measured using the Mann–Whitney U test. We used the Bonferroni method to account for multiple comparisons. Backward linear regression analysis was performed to investigate predictors of glutamine levels at admission.

A two-sided *P* value < 0.05 was considered significant. All tests of the data were performed using Statistical Package for the Social Sciences 21.0 (IBM, New York, NY, USA).

## Results

The patients’ characteristics are shown in Table 1. We included 88 patients in the elective surgery group (cardiac surgery [*n* = 76] and general surgery [*n* = 12]) and 90 patients in the non-elective group (sepsis [*n* = 27], acute surgery [*n* = 17], trauma [*n* = 2], and medical [*n* = 44]). Patients in the elective group were significantly older (*P* < 0.001) and more often male (*P* < 0.001) than those in the non-elective group. The Acute Physiology and Chronic Health Evaluation (APACHE) IV score was significantly higher in the non-elective group than in the elective group (*P* < 00.1). Non-elective patients stayed longer in the ICU, but there was no significant difference in hospital stay between the groups. One patient in the elective group died, whereas in the non-elective group, seven patients did not survive.

**Table 1:** Patients’ characteristics at admission

	Elective (*N* = 88)	Non-elective (*N* = 90)	*P* value
Age (years)	68 ± 10	59 ± 17	<0.001
Male/female	65 / 33	46 / 44	<0.001
Length (cm)	175 ± 8	174 ± 10	0.25
Weight (kg)	83 ± 13	82 ± 18	0.67
BMI	27 ± 4	27 ± 6	0.71
APACHE IV	46 ± 14	67 ± 29	<0.001
LOS ICU (days)	2 [2–2]	4 [2–7]	<0.001
LOS Hospital (days)	6 [4–9]	8 [3–13]	0.71
Survival ICU (%)	100	94	0.01
Survival hospital (%)	99	92	0.02
Laboratory measurements:			
CRP mg/l	20 [7–71]	35 [5–171]	0.49
Creatinine μmol/l	87 [71–101]	74 [56–107]	0.08
AF U/l	75 [49–87]	73 [51–107]	0.47
CK U/l	249 [157–474]	90 [47–197]	<0.001

Values are mean ± standard deviation or median [interquartile range]

*APACHE* Acute Physiology and Chronic Health Evaluation, *LOS* length of stay, *CRP* C-reactive protein, *AF* alkaline phosphatase, *CK* creatine kinase

The median plasma glutamine level at ICU admission was significantly higher in elective patients compared with non-elective patients (0.43 mmol/l [0.33–0.55 mmol/l] versus 0.25 mmol/l [0.09–0.37 mmol/l], *P* = 0.001). During the ICU stay, plasma glutamine levels remained higher between elective and non-elective patients, but they tended to decrease in the elective group as they stayed longer in the ICU (Table 2).

**Table 2:** Plasma glutamine levels (mmol/l) at admission, and on days 1 and 2 after admission

	Elective	Non-elective	*P*
Glutamine at admission	0.43 [0.33–0.55] *n* = 88	0.25 [0.09–0.37] *n* = 90	<0.001
Glutamine at day 1	0.42 [0.24–0.55] *n* = 62	0.21 [0.09–0.33] *n* = 76	<0.001
Glutamine at day 2	0.34 [0.21–0.57] *n* = 11	0.20 [0.08–0.37] *n* = 46	0.08

Values are median [interquartile range] and the number of patients (*n*)

A total of 112 (65 %) patients had a plasma glutamine level lower than 0.420 mmol/l at ICU admission. Significantly less elective patients showed a plasma glutamine level lower than 0.420 mmol/l than non-elective patients (34 vs 74 %, *P* < 0.001).

We divided the non-elective patients into those with and without infection. In patients with infection (*n* = 27), glutamine levels were significantly lower than those in patients without infection (Table 3).

**Table 3:** Plasma glutamine levels (mmol/l) in the non-elective group, with or without infection

	Infection	No infection	*P* value
Glutamine at admission	0.01 [0.00–0.16] *n* = 27	0.28 [0.17–0.41] *n* = 63	<0.001
Glutamine at day 1	0.08 [0.00–0.24] *n* = 25	0.27 [0.16–0.37] *n* = 51	<0.001
Glutamine at day 2	0.09 [0.01–0.19] *n* = 16	0.25 [0.14–0.42] *n* = 30	0.01

Values are median [interquartile range] and the number of patients (*n*)

There appeared to be a significant correlation between the APACHE IV score and glutamine levels (*R* = 0.52, *P* < 0.001). Moreover, in backward linear regression analysis, this correlation was independently associated with the APACHE IV score and the presence of infection, but not with the type of admission.

## Discussion

In the present study, we found that plasma glutamine levels were significantly lower in patients with non-elective admission to the ICU compared with those who were admitted after elective surgery. Inflammation caused by elective surgery can lead to a decrease in glutamine levels, but this is less pronounced compared with other forms of acute critical illness. Patients who are admitted after elective surgery are unlikely to have an infection at the time of admission to the ICU. This could have played a role in the differences that were found in plasma glutamine levels between the two groups. Additionally, the time between the onset of a critical illness and admission to an ICU is generally longer than the time between the start of surgery and admission. Therefore, a longer period of catabolic stress might lead to a further decrease in glutamine levels.

In our study, in the non-elective group, 74 % of patients had a glutamine level lower than 0.420 mmol/l. The percentage of patients with a low glutamine level in our study is higher than that reported in previous studies [3–5]. Additionally, a considerable amount of patients after elective surgery had a glutamine level below 0.42 mmol/l. We did not measure plasma glutamine levels before surgery. Therefore, whether plasma glutamine levels were already low before surgery in these patients or whether lowering plasma glutamine levels was a consequence of surgery is unknown. Most of the patients in the elective surgery group underwent cardiac surgery with cardiopulmonary bypass. Extracorporeal circulation induces an inflammatory response, but there are no clinical data on the effects on plasma glutamine levels in this situation [11, 12]. In a small series of patients, a decrease in glutamine levels was reported after elective abdominal aneurysm surgery [13].

We also measured plasma glutamine levels on days 1 and 2 of the ICU stay. During these days, the difference in plasma glutamine levels between non-elective and elective admission remained significant. As expected, only a few patients stayed longer in the ICU after elective surgery. In general, these were patients with a more complex postoperative course, and this could explain the decline in plasma glutamine levels during their ICU stay.

In non-elective patients with an infection, plasma glutamine levels were significantly lower than those in non-elective patients without an infection. Additionally, plasma glutamine levels were independently associated with the presence of an infection. These findings accentuate the role of inflammation in plasma glutamine levels.

In contrast to previous studies, we found that glutamine levels were determined by the severity of illness as expressed by the APACHE IV score [3, 4]. If plasma glutamine levels are solely considered as an additional risk factor, there is no additional advantage for glutamine over the well-calibrated APACHE IV model. However, with regard to organ dysfunction in pro-inflammatory disease states, our finding adds to the belief that glutamine levels may play a role in the aetiology. In two previous studies, there appeared to be no relation between plasma glutamine levels and the APACHE II score or sequential organ failure assessment score, but they were significantly related to mortality [3, 4]. Mortality in the present study was so low that we did not find an association with plasma glutamine levels. Interestingly, in a study by Rodas et al., the association of plasma glutamine levels with mortality only became apparent at 6 months after discharge from the ICU [4].

During the last decades, ICU patients have been treated with glutamine because available evidence has shown a benefit in survival when glutamine was provided intravenously to these patients [14]. Recently, the use of glutamine is under debate because two large randomised controlled trials in which critically ill patients were treated with enteral and intravenous glutamine did not show a benefit in survival [15, 16]. Whether low plasma glutamine levels during critical illness reflect an adequate response to stress or reveal a deficiency in the amount of glutamine that can be used is unclear [17]. Therefore, whether a low plasma glutamine level is a condition that should be treated is unknown. Glutamine is involved in many cellular functions, but its precise role during catabolic stress is not completely understood.

In all clinical trials that have been published on glutamine supplementation, glutamine was provided without consideration of plasma glutamine levels in patients who were studied [14–16]. To determine whether supplementation of glutamine is beneficial for ICU patients, we should only treat patients with low plasma glutamine levels. The method that was used in the present study enabled us to obtain plasma glutamine measurements soon after blood sampling. Using this method means that patients with low plasma glutamine levels early in the course of critical illness can be identified before considering supplementation. Future studies need to determine whether glutamine supplementation is beneficial in patients with low glutamine levels at ICU admission or during the ICU stay.

## Conclusion

Plasma glutamine levels are significantly lower after non-elective ICU admission compared with elective ICU admission. A considerable amount of patients have decreased plasma glutamine levels, but this is not independently associated with the type of admission. In contrast to previous studies, we found that plasma glutamine levels were determined by the severity of illness and presence of an infection.

## Key messages

- Plasma glutamine levels are significantly lower after non-elective ICU admission compared with elective ICU admission.
- Plasma glutamine levels are not associated with the type of admission.
- Plasma glutamine levels are determined by the severity of illness (APACHE IV) and the presence of an infection.

## Additional file

Additional file 1: Glutamine measurements. Measurement of glutamine was performed with the Bioprofile 100 plus analyser (Nova Biomedical U.K., Cheshire, UK). This analyser was originally intended for monitoring metabolites and nutrients in cell culture systems, and was used in this study to monitor glutamine in plasma samples (plasma separator tubes; Becton Dickinson B.V., Breda, the Netherlands). For pre-validation of the analyser, we used water-based controls from the supplier of this analyser, and the measurements appeared reproducible. Control measurements were as follows: level 1 mean (± standard deviation) was 1.06 ± 0.061 mmol/l, the CV (coefficient of variation) was 5.7 %, and *n* = 143; level 2 mean was 5.31 ± 0.400 mmol/l, the CV was 7.5 %, and *n* = 152. Using home-made serum/plasma controls, we obtained the following results: heparin pool 1, 0.558 ± 0.056 mmol/l, CV = 10.1 %, *n* = 45; serum pool, 1.038 ± 0.086 mmol/l, CV = 8.3 %, *n* = 45; and heparin pool 2, 5.279 ± 0.259 mmol/l, CV = 4.9 %, *n* = 42. We established that plasma glutamine levels were stable when plasma was separated from cells within 3 h after sampling and frozen until analysis (or measured within 3 h after plasma preparation) (Figure S1). No significant differences in plasma glutamine levels were observed in outpatients (mean difference [direct measurement versus measurement after 24 h at −20 °C]: −0.013 mmol/L; 95 % confidence interval: −0.035 – −0.010; two-sided *P* = 0.2675, *n* = 20) and in patients with sepsis (mean difference [direct measurement versus measurement after 24 h at −20 °C]: −0.022 mmol/L; 95 % confidence interval: −0.039 – −0.005; two-sided *P* = 0.0137, *n* = 20). In outpatients (*n* = 51), we obtained a plasma glutamine reference interval, which ranged from 0.22–0.59 mmol/l (Figure S2). (PDF 303kkb)

## Abbreviations

AF: alkaline phosphatase
APACHE: Acute Physiology and Chronic Health Evaluation
BMI: body mass index
CK: creatine kinase
CRP: C-reactive protein
ICU: intensive care unit
LOS: length of stay
SD: standard deviation
