# Supplementary material for: Plasma glutamine levels in patients after non-elective or elective ICU admission: an observational study
Source: BMC Anesthesiol. 2016 Mar 10;16:15. doi: 10.1186/s12871-016-0180-7 (PMC4787198; doi:10.1186/s12871-016-0180-7)
Supplement: Additional file 1: — Glutamine measurements. Measurement of glutamine was performed with the Bioprofile 100 plus analyser (Nova Biomedical U.K., Cheshire, UK). This analyser was originally intended for monitoring metabolites and nutrients in cell culture systems, and was used in this study to monitor glutamine in plasma samples (plasma separator tubes; Becton Dickinson B.V., Breda, the Netherlands). For pre-validation of the analyser, we used water-based controls from the supplier of this analyser, and the measurements appeared reproducible. Control measurements were as follows: level 1 mean (± standard deviation) was 1.06 ± 0.061 mmol/l, the CV (coefficient of variation) was 5.7 %, and n = 143; level 2 mean was 5.31 ± 0.400 mmol/l, the CV was 7.5 %, and n = 152. Using home-made serum/plasma controls, we obtained the following results: heparin pool 1, 0.558 ± 0.056 mmol/l, CV = 10.1 %, n = 45; serum pool, 1.038 ± 0.086 mmol/l, CV = 8.3 %, n = 45; and heparin pool 2, 5.279 ± 0.259 mmol/l, CV = 4.9 %, n = 42. We established that plasma glutamine levels were stable when plasma was separated from cells within 3 h after sampling and frozen until analysis (or measured within 3 h after plasma preparation) (Figure S1). No significant differences in plasma glutamine levels were observed in outpatients (mean difference [direct measurement versus measurement after 24 h at −20 °C]: −0.013 mmol/L; 95 % confidence interval: −0.035 – −0.010; two-sided P = 0.2675, n = 20) and in patients with sepsis (mean difference [direct measurement versus measurement after 24 h at −20 °C]: −0.022 mmol/L; 95 % confidence interval: −0.039 – −0.005; two-sided P = 0.0137, n = 20). In outpatients (n = 51), we obtained a plasma glutamine reference interval, which ranged from 0.22–0.59 mmol/l (Figure S2). (PDF 303kkb) [file 12871_2016_180_MOESM1_ESM.pdf]

Additional file 1

Glutamine measurements

Measurement of glutamine was performed with the Bioprofile 100 plus analyser (Nova Biomedical U.K., Cheshire, UK). This analyser was originally intended for monitoring metabolites and nutrients in cell culture systems, and was used in this study to monitor glutamine in plasma samples (plasma separator tubes; Becton Dickinson B.V., Breda, the Netherlands). For pre-validation of the analyser, we used water-based controls from the supplier of this analyser, and the measurements appeared reproducible. Control measurements were as follows: level 1 mean (± standard deviation) was 1.06 ± 0.061 mmol/l, the CV (coefficient of variation) was 5.7%, and n = 143; level 2 mean was 5.31 ± 0.400 mmol/l, the CV was 7.5%, and n = 152. Using home-made serum/plasma controls, we obtained the following results: heparin pool 1, 0.558 ± 0.056 mmol/l, CV = 10.1%, n = 45; serum pool, 1.038 ± 0.086 mmol/l, CV = 8.3%, n = 45; and heparin pool 2, 5.279 ± 0.259 mmol/l, CV = 4.9%, n = 42. We established that plasma glutamine levels were stable when plasma was separated from cells within 3 h after sampling and frozen until analysis (or measured within 3 h after plasma preparation) (Fig. 1). No significant differences in plasma glutamine levels were observed in outpatients (mean difference [direct measurement versus measurement after 24 h at −20°C]: −0.013 mmol/L; 95% confidence interval: −0.035 to −0.010; two-sided P = 0.2675, n = 20) and in patients with sepsis (mean difference [direct measurement versus measurement after 24 h at −20°C]: −0.022 mmol/L; 95% confidence interval: −0.039 to −0.005; two-sided P = 0.0137, n = 20).

In outpatients (n = 51), we obtained a plasma glutamine reference interval, which ranged from 0.22–0.59 mmol/l (Fig. 2).

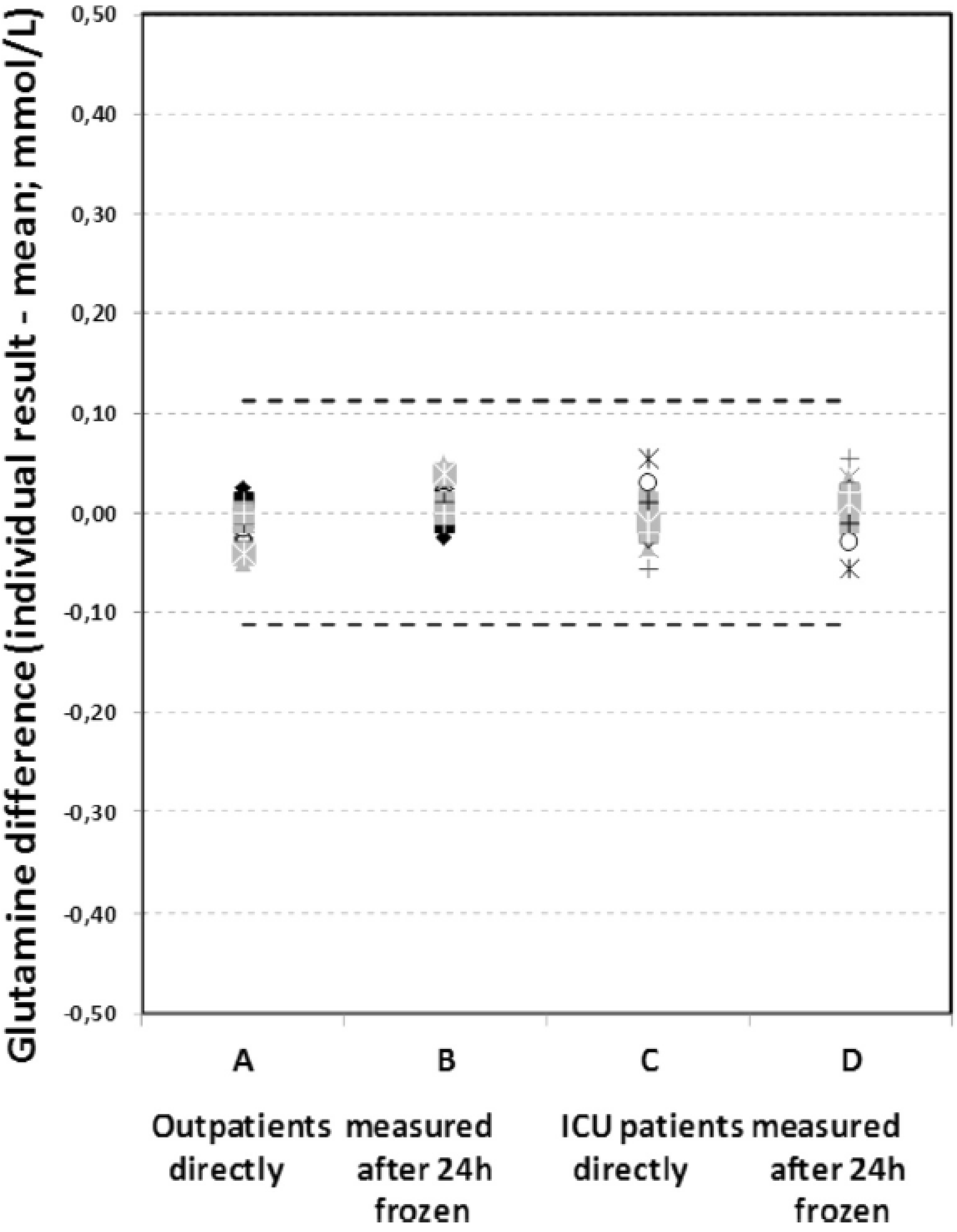

Fig. 1 Stability of glutamine in plasma. Plasma was separated from cells within 3 h after collection, frozen immediately thereafter, and measured 24 h later.

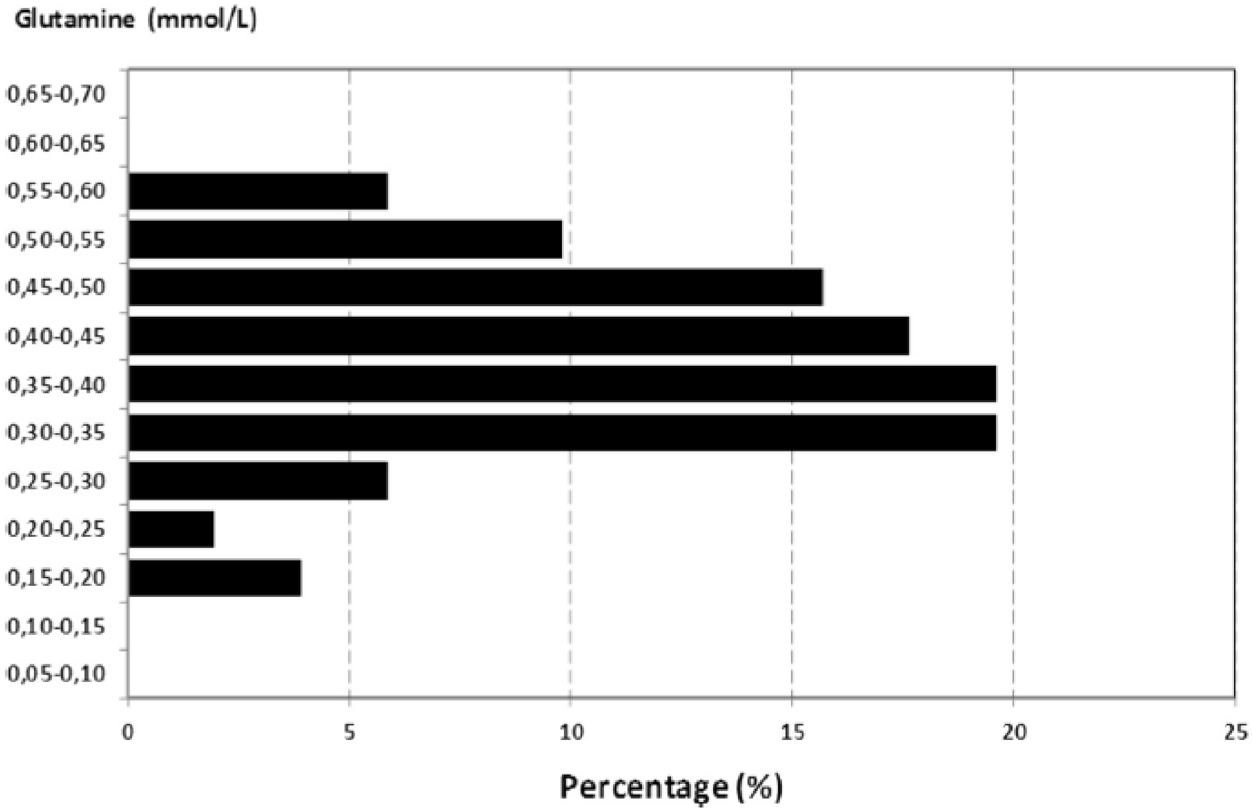

Fig. 2 Histogram of glutamine values found in 51 outpatients
